# AXL kinase as a novel target for cancer therapy

**DOI:** 10.18632/oncotarget.2542

**Published:** 2014-10-16

**Authors:** Xiaoliang Wu, Xuewen Liu, Sanjay Koul, Chang Youl Lee, Zhenfeng Zhang, Balazs Halmos

**Affiliations:** ^1^ Sun Yat-sen University Cancer Center, State Key Laboratory of Oncology in South China, Collaborative Innovation Center for Cancer Medicine, Guangzhou, People’s Republic of China; ^2^ Division of Hematology/Oncology, Herbert Irving Comprehensive Cancer Center, New York Presbyterian Hospital-Columbia University Medical Center, New York, NY, USA; ^3^ Division of Pulmonary, Allergy and Critical Care Medicine, Department of Internal Medicine, Chuncheon Sacred Heart Hospital Hallym University Medical Center, Chuncheon-si Gangwon-do 200–704 Republic of Korea

**Keywords:** AXL, receptor tyrosine kinase, lung cancer, targeted therapy

## Abstract

The AXL receptor tyrosine kinase and its major ligand, GAS6 have been demonstrated to be overexpressed and activated in many human cancers (such as lung, breast, and pancreatic cancer) and have been correlated with poor prognosis, promotion of increased invasiveness/metastasis, the EMT phenotype and drug resistance. Targeting AXL in different model systems with specific small molecule kinase inhibitors or antibodies alone or in combination with other drugs can lead to inactivation of AXL-mediated signaling pathways and can lead to regained drug sensitivity and improved therapeutic efficacy, defining AXL as a promising novel target for cancer therapeutics. This review highlights the data supporting AXL as a novel treatment candidate in a variety of cancers as well as the current status of drug development targeting the AXL/GAS6 axis and future perspectives in this emerging field.

## Introduction to the TAM tyrosine kinase receptor family

**Figure 1:**
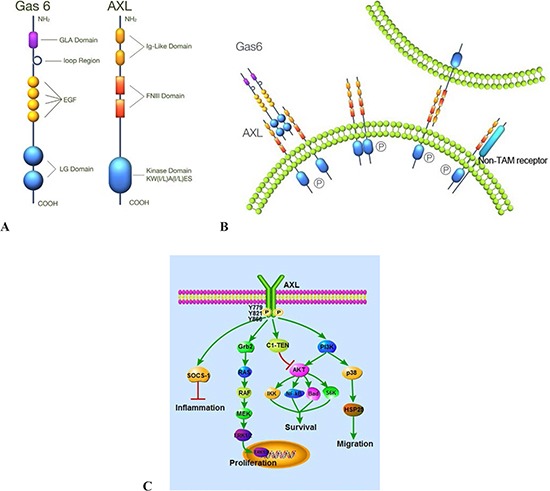
Structure, activation and signaling pathways of AXL **(A)** AXL consists of two immunoglobulin-like (Ig) domains and dual fibronectin type III (FNIII) repeat domains and a kinase domain. Gas6 contains a γ-carboxyglutamic acid (Gla) domain, a loop region, four EGF-like repeats and two C-terminal globular laminin G-like (LG) domains. **(B)** AXL can be activated by ligand-dependent dimerization, ligand-independent dimerization, and interaction between two monomers on neighboring cells and heteromeric dimerization with a non-TAM receptor. **(C)** AXL plays important roles in cell proliferation, survival, migration, and the inflammatory process via different signaling pathways.

The tyrosine kinase family of proteins is composed of two major groups, receptor tyrosine kinases (RTKs) and non-receptor tyrosine kinases (NRTKs). RTKs are well known to be involved in tumorigenesis and many of these serve as actionable targets for cancer therapy. The TAM group of RTKs is a recently identified class of the RTK subfamily that transduces crucial extracellular signals to the inside of the cell [1]. The small family of TAM receptor kinases include TYRO-3 (also known as Brt, Dtk, Rse, Sky and Tif), AXL (also known as Ark, Tyro7 and Ufo), and MER (also known as Eyk, Nym and Tyro12) [2, 3]. The transforming gene, AXL (derived from the Greek word “anexelekto”, meaning uncontrolled) was originally isolated from chronic myelogenous leukemia cells [4]. The AXL gene is located on chromosome 19q13.2 and encodes 20 exons [5]. The MER and TYRO-3 genes are located on chromosome 2q 14.1 and chromosome 15q15, respectively. The TAM family is characterized by a combination of two immunoglobulin-like (Ig) domains and dual fibronectin type III (FNIII) repeat domains in the extracellular region, a transmembrane domain and a cytoplasmic tyrosine kinase domain (Figure 1A) [2, 6].

## AXL ligands

The TAM family kinases were initially considered to be orphan receptors [4, 7] but now it is understood that there are diverse ligands for this family of receptors. Growth arrest specific gene 6 (Gas6), protein S, Tubby, Tubby-like protein 1 (Tulp-1) and Galectin-3 are known ligands for TAM family members. Gas6 and protein S are members of the vitamin K-dependent protein family [8–10]. Gas6 cDNA shows significant homology to protein S [9, 11] and both are secreted proteins and mediate their action through binding to and activating AXL, Tyro3 and Mer [12]. Gas6 and protein S have different receptor-binding specificity. Gas6 binds to all three TAM RTKs (AXL>TYRO-3>MER), whereas protein S interacts only with MER and TYRO-3 but not AXL [13–17]. Gas6 has 3- to 10-fold higher affinity for AXL than MER. In addition, several reports suggest that Tubby, Tulp-1 and Galectin-3 are also novel ligands for TAM receptors. Similar to Gas6 and protein S, tubby and tulp-1 have distinct binding specificities to TAM RTKs. Tulp-1 bind to all three RTKs, whereas Tubby only recognizes MER [18, 19].

## AXL signaling: activation and regulation

AXL can be activated through a number of different mechanisms: ligand-dependent dimerization (principally driven by Gas-6), ligand-independent dimerization, interaction between two monomers on neighboring cells and heteromeric dimerization with a non-TAM receptor (Figure 1B) [3, 12, 13, 20]. Gas6-mediated AXL dimerization is likely to occur in two steps, with a high-affinity 1:1 Gas6/AXL complex forming first, then lateral diffusion of such complexes leading to the formation of a dimeric signaling complex [6]. Gas6 binding to the extracellular domain of AXL leads to autophosphorylation of tyrosine residues on the intracellular tyrosine kinase domain of AXL, including Y779, Y821, Y866 (Figure 1C) [21]. Gas6/AXL signaling plays diverse roles in numerous cellular activities [22]. These effects are mainly mediated by Gas6/AXL-induced activation of MAPK/ERK and PI3K/AKT signaling pathways. C1-TEN and SOCS-1 have been identified as negative regulators of AXL signaling. In particular, C1-TEN can negatively regulate AXL-mediated PI3K/AKT signaling and thereby reduces cell survival, proliferation, and migration of HEK293 cells (Figure 1C) [23, 24]. In murine and human plasma, soluble forms of AXL (sAXL) are produced by proteolytic cleavage and sAXL binds to Gas6 thereby inhibiting cellular activation of AXL [25].

## Physiological roles of TAM receptors

The TAM family of RTKs regulates an intriguing mix of physiological processes, including cell proliferation, survival, cell adhesion and migration, blood clot stabilization, and regulation of inflammatory cytokine release. Although expression of TAM receptor mRNAs is observed in embryonic tissues [26–28], single, double, and even triple knockouts are viable without any obvious signs of developmental defects at birth [29–31], suggesting that the TAM RTKs are nonessential for embryogenesis[32]. Conversely, adult TAM knockout mice develop diverse phenotypes in a wide range of tissues revealing prominent cellular functions for TAM receptors. In adult tissues, TYRO-3, AXL, and MER exhibit widespread distribution with overlapping but unique expression profiles. In adult tissues, AXL is expressed ubiquitously[4], with high levels found in hippocampus and cerebellum, platelets, monocytes/macrophages, endothelial cells, skeletal muscle, heart, kidney, testes, liver, as well as in different cell lines of mesenchymal, epithelial and hematopoietic origin [10, 33–35].

## AXL expression in cancer

AXL has been reported to be overexpressed or ectopically expressed in a multitude of cancers (Table 1). While overexpression in general is defined as expression higher than normal tissue counterparts, it needs to be highlighted that the listed manuscripts vary greatly in their approach as to this definition and there is a need for the development of a validated, reliable assay to measure AXL expression. AXL gene expression is regulated by Sp1/Sp3 transcription factors. In addition, methylation of CpG sites within specific Sp1 motifs of the promoter can also modulate AXL gene expression [36]. Myeloid zinc finger 1 (MZF1), a SCAN domain family transcription factor, can also bind to the AXL promoter and transactivate its expression [37]. In addition, specific miRs (microRNAs) that target the 3′-UTR of the AXL gene have been identified in several cancer cell lines. AXL receptor expression can specifically be regulated by miR-34a and miR-199a/b, which are suppressed by promoter methylation in a number of solid tumors (such as non-small cell lung cancer, breast cancer, colorectal cancer) [38].

**Table 1:** AXL Overexpression or ectopic expression in a multitude of cancers

Tumors	Cell lines/Tissue	References
Lung cancer	cell lines and tissue	[39–41]
Breast cancer	cell lines and tissue	[42, 43]
Myeloid leukemia	cell lines	[44–46]
Chronic Lymphocytic Leukemia	cell lines	[47]
Colon cancer	cell lines and tissue	[37, 48]
Esophageal adenocarcinoma	cell lines and tissue	[49, 50]
Gastric cancer	cell lines and tissue	[51]
Ovarian cancer	cell lines and tissue	[52, 53]
Hepatocellular carcinoma	cell lines and tissue	[54, 55]
Prostate cancer	cell lines and tissue	[56–58]
Pancreatic adenocarcinoma	cell lines and tissue	[59, 60]
Thyroid cancer	cell lines and tissue	[61, 62]
Glioma	cell lines and tissue	[63–65]
Renal cell carcinoma	cell lines and tissue	[66, 67]
Melanomas	cell lines and tissue	[68–70]
Malignant pleural mesothelioma	tissue	[71]
Cutaneous squamous cell carcinoma	cell lines and tissue	[72]
Endometrial cancers	cell lines and tissue	[73]

## AXL overexpression in lung cancer

Overexpression of AXL has been observed in approximately 60% of NSCLC cell lines, and AXL is also highly expressed in a significant fraction of primary lung cancers [40, 74]. It has been shown that in the H1299 lung cancer cell line expressing different mutant forms of p53, AXL is upregulated upon p53 loss and at least in part gain-of-function activities of mutant p53 were mediated through AXL overexpression as knockdown of AXL by AXL-specific siRNA reduced cell growth and motility [75]. Rac1 has been identified as a downstream effector of AXL via the PI3K/AKT pathway promoting reactive oxygen species (ROS) production and cell migration. Excessive ROS may in turn lead to AXL phosphorylation and Akt1 and Rac1 activation may play important roles in cell migration under oxidative stress[76]. Depletion of Rac1 in NSCLC cells was found to result in decreased anticancer drug resistance and inhibited rearrangements of the actin cytoskeleton that attenuated cell migration [77, 78]. Yes-associated protein 1 (YAP1) is a nuclear effector of the Hippo pathway that plays an important role in tumorigenesis and progression in lung adenocarcinomas. Compared to normal lung tissue, YAP1 was shown overexpressed in lung adenocarcinomas, and knockdown of YAP1 markedly downregulated the expression of AXL and inhibited the proliferation and invasion of lung adenocarcinomas suggesting that AXL is a mediator of the oncogenic function of YAP1 [79].

## AXL in breast cancer

AXL is overexpressed in highly invasive breast cancer cell lines (such as Hs578T, BT549, MDA-MB-435s, MDA-MB-157, MDA-MB-436, and MDA-MB-231); in contrast, weakly invasive breast cancer cell lines do not or only weakly express AXL. It has also been demonstrated that AXL expression correlates with motility and invasiveness in breast cancer cells [42]. The miR-34a/AXL interaction was functionally characterized through ectopic overexpression experiments with a miR-34a mimic in two independent triple-negative breast cancer cell lines. MiR-34a was found to bind to its putative target site within the AXL 3′-UTR to regulate AXL expression and impair its functions [38, 80]. Studies suggest that the estrogen receptor can induce AXL expression and this interaction could play an important role in the proliferation, differentiation and apoptotic cell death in human breast epithelium [81]. Gas6 has been demonstrated to be upregulated more than 20-fold through the progesterone receptor B (PRB) [82], and Her-2 is also reported to trigger AXL activation, suggesting crosstalk with this clinically important pathway [83]. Tazarotene-induced gene 1 (TIG1) is highly expressed in inflammatory breast cancer, promoting tumor growth and invasion. It appears that TIG1 physically associates with and stabilizes AXL protein in vivo by inhibiting its proteasomal degradation. These data also strongly suggest that AXL might be an important therapeutic target in inflammatory breast cancer [43].

## AXL in AML

AXL was found to be an independent prognostic marker and therapeutic target in acute myeloid leukemia (AML). It appears that AML cells can induce expression and secretion of Gas6 from bone marrow derived stromal cells and Gas6 in turn mediates proliferation, survival and chemoresistance of AXL expressing AML cells. This paracrine axis creates a chemoprotective tumor cell niche. AXL inhibition with the small molecule inhibitor, BGB324 is able to overcome this mechanism and can synergize with chemotherapy. In this study, 57% of AML patient samples showed AXL expression and overall outcome in this subset was worse. 90% of samples showed Gas6 expression, however further analysis showed that this expression was predominantly related to stromal expression [44].

AXL in prostate and pancreatic cancer

Increased AXL expression was demonstrated to promote migration and invasion of prostate cancer cells in vitro [56, 57, 84], and is also associated with a higher frequency of distant metastasis after pancreaticoduodenectomy in patients with pancreatic adenocarcinoma [59, 60, 85].

## AXL as a potential oncogenic gene involved in EMT and metastasis/invasion

The epithelial–mesenchymal transition (EMT) describes a reversible switch from an epithelial-like to a mesenchymal-like phenotype and it is a global program that endows epithelial cells with the ability to migrate and invade surrounding tissue. It is essential for the development of normal epithelium, and also contributes to the invasive and metastatic properties of carcinomas [86, 87]. In a number of cancers, development of acquired resistance correlates with EMT traits, such as metastatic potential, invasiveness and mesenchymal-like traits. For example, our group was the first to demonstrate that EGFR TKI-resistant NSCLC cells not only gained AXL upregulation, but also demonstrated a concomitant EMT phenotype [41]. Other recent studies also showed that AXL expression was associated with EMT and therapeutic resistance may be associated with histological changes [88, 89]. EMT-associated up-regulation of AXL can drive autocrine interactions with Gas6 produced by endothelial cells, suggesting that autocrine AXL signaling might be a frequent consequence of EMT in many tumor types [90, 91]. Further cell line models have demonstrated that AXL mRNA is induced in HCC4006, HCC2279, H1650, and H1975 cells following the development of acquired resistance to EGFR-specific TKIs and this was again noted to be associated with an EMT program switch [92, 93]. Furthermore, two studies show that AXL is activated in breast cancer stem cells (BCSC) and AXL expression induces EMT through direct regulation of the expression of E-cadherin, N-cadherin, Slug and Snail. AXL-driven EMT induction regulates BCSC invasion, migration and chemoresistance. Treatment with the AXL inhibitor MP470 (Amuvatinib) reversed EMT and thereby reduced tumor growth, downregulated NF-kB pathway activity and led to restoration of chemosensitivity in murine BCSCs and mesenchymal normal human mammary epithelial cells [94, 95].

In order to reveal the actual mechanisms underlying the association of AXL and EMT, many factors related to EMT have been explored. For example, vimentin expression was shown to be required for the induction of AXL expression and such regulation functionally contributed to the EMT phenotype in breast cancer cells and accordingly silencing of vimentin decreased AXL levels significantly [89]. MZF1 was demonstrated to bind to the AXL promoter and induced invasion and metastasis in colorectal and cervical cancer cells, at least in part by regulating AXL gene expression [37]. TAZWW domain containing transcription regulator 1(TAZ) promotes EMT-mediated cancer progression [96–98] and TAZ-AXL-CTGF overexpression was associated with increased expression of genes that were associated with colon cancer progression, potentially serving as a novel prognostic indicator for colon cancer progression [99]. Recently, AXL has been demonstrated as a key regulator of inherent and chemotherapy-induced invasion in invasive or migratory CRC cell subpopulations with an EMT-like phenotype and increased AXL levels predict a poor clinical outcome in early stage colon cancer[100]. The important role of AXL in EMT might seem contradictory in face of the lack of an embryonic phenotype and this might possibly be explained by compensatory mechanisms that might specifically exist in embryonic tissues.

## AXL and drug resistance

Several studies suggest that overexpression of AXL may result in resistance to both targeted therapies and conventional chemotherapy in different models, such as lung cancer [41], breast cancer [101], esophageal carcinoma [49], gastrointestinal stromal tumors [102], and AML [103]. In our recent study, we independently established new cellular models of acquired resistance to the EGFR TKI erlotinib in EGFR-mutated lung adenocarcinoma by using the erlotinib-sensitive, EGFR-mutated NSCLC cell line HCC827, and revealed that increased activation of AXL and induction of an EMT-like state was associated with acquired resistance to erlotinib in vitro and in vivo, identifying AXL as a promising therapeutic target to prevent or overcome resistance. AXL inhibition via AXL knockdown studies or through the use of AXL tyrosine kinase inhibitors was able to reverse acquired resistance and 20% of primary tumor samples demonstrated significant upregulation of AXL upon the development of in vivo resistance as compared to pre-treatment samples [41, 104]. Another study looked at a uniform group of 26 patients from one referral center in Korea for mechanisms of acquired resistance and similarly found a 19% frequency of AXL overexpression upon acquired resistance to gefitinib (5/26). In one of these patients the tumor also harbored an EGFR-T790M resistance mutation [105]. More recently, a novel MET and AXL inhibitor NPS-1034 was reported to exert efficacy against NSCLC cells resistant to EGFR-TKIs due to MET or AXL activation [106]. While the mechanism of AXL overexpression upon acquired resistance remains unclear in vivo, one study suggests that hypomethylation of the AXL promoter may play a role in AXL overexpression in ErbB2-positive breast cancer cells upon acquisition of resistance to the ErbB2 inhibitor, lapatinib. In addition, the multikinase inhibitor GSK1363089 that also targets AXL can restore lapatinib sensitivity in HER2-positive breast cancer cells that overexpress AXL [101]. Analogous to our findings with erlotinib resistance, at least in vitro crizotinib resistance in ALK-translocation positive lung cancer models also appears to be mediated by an EMT transition accompanied by AXL overexpression. However, in this model AXL inhibition could not overcome resistance to crizotinib but cellular migration and invasion were both reduced [107]. Furthermore, AXL and its ligand Gas6 were similarly observed to be upregulated along with vimentin upregulation and E-cadherin loss in the COR cell clones resistant to the EGFR T790M-targeting drug, CO-1686 in EGFR-mutated lung adenocarcinoma [108]. Further corroborating data suggesting the importance of AXL as a resistance marker came from a recent study [93] that identified a 76 gene expression signature of EMT that can classify NSCLCs either as epithelial or mesenchymal. This signature with AXL expression as its prime marker was found to be a biomarker of response to targeted therapies, such as erlotinib or PI3kinase inhibitor therapy. Pharmacological inhibition of AXL in some of the models demonstrated synergistic effects with erlotinib. This was then clinically validated by using samples from the so-called BATTLE study. These data suggest that AXL inhibition might enhance responses to erlotinib in some NSCLC patients with wild-type EGFR as well. Prospective clinical studies incorporating AXL expression as a predictive biomarker of drug response and a novel therapeutic target are warranted. These data also suggest that inhibiting AXL signaling in NSCLC with a mesenchymal phenotype might have therapeutic value.

Recently, expression of AXL has been identified as a predictor for lack of response to EGFR–targeted inhibitors in triple-negative breast cancer cells[109]. In this very interesting study a unique machine learning analysis of the CCLE database was performed and elevated AXL gene expression was found to be exceptionally predictive of lack of response to ErbB family inhibitors, such as lapatinib and erlotinib. It was also found that EGFR signaling transactivates AXL and this ligand-independent AXL activity could then diversify downstream signaling pathways beyond those triggered by EGFR alone. Co-IP studies also showed that AXL physically associates with EGFR, other ErbB members as well as MET and PDGFR. Triple negative breast cancer (TNBC) cell lines that overexpress both EGFR and AXL appear to be more sensitive to AXL than EGFR inhibition. This is particularly relevant as TBNs are known to have high expression of EGFR; still EGFR inhibitors have not demonstrated sufficient activity by themselves in this highly aggressive histotype. Interestingly, combined inhibition of EGFR and AXL was subadditive and this might be explained by the fact that they utilize shared downstream pathway components[95]. Indeed, activation of EGFR by EGF stimulation of MDA-MB-231 cells led to both MET and AXL phosphorylation but not vice versa. EGFR-mediated AXL activation led to widespread downstream signaling changes blocked by AXL siRNA, while Gas6 stimulation had lesser effects. This pivotal study overall suggests that AXL can serve as an amplifier of EGFR signaling – at least when it comes to AKT activation. In addition, AXL can activate a set of distinct signals that are important for cell migration in response to stimuli and RTK/AXL co-localization can predict RTK-mediated AXL transactivation leading to downstream pathway output amplification. Based on this and other studies it appears that ErbB, MET and AXL receptors can co-exist in local clusters on the plasma membrane leading to subsequent activation-dependent enhancement of interactions after stimulation. It might be anticipated that drugs disrupting this complex interaction may be efficacious in counteracting such signal diversification.

Overexpression of AXL in esophageal carcinoma has been shown to promote cisplatin resistance through regulation of c-ABL/p73 signaling [49]. In AML, AXL has been shown to be induced by various chemotherapeutic drugs [103], suggesting that a combination of AXL inhibition with chemotherapy may be a novel therapeutic approach in AML [44, 110]. AXL overexpression and/or activation have also been linked to Nilotinib resistance in chronic myeloid leukemia (CML) cells, and AXL expression could be a clinically relevant prognostic marker for resistance to TKI therapy in CML cells [45, 46]. AXL was also found to be highly expressed in cisplatin-resistant ovarian cancer and was identified as an essential factor and therapeutic target in metastatic ovarian cancer [53, 111, 112]. These results suggest that AXL can promote resistance not only to tyrosine kinase inhibitors but more broadly as well.

## Interplay of AXL/MET and HER family receptors

**Figure 2:**
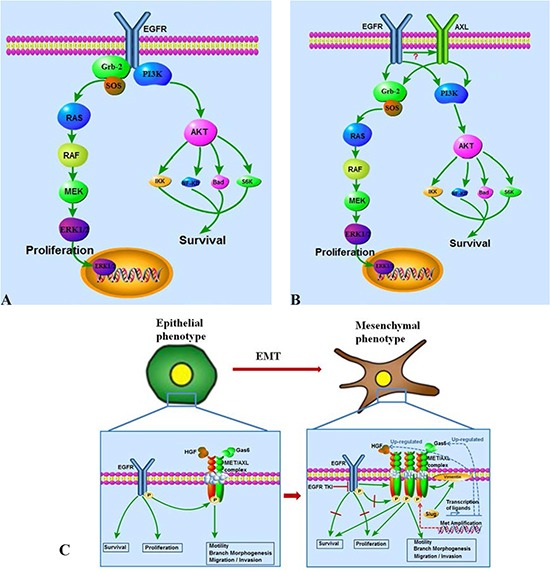
EGFR and AXL/MET switch plays critical roles in acquired EGFR TKI resistance and correlated epithelial-mesenchymal transition (EMT) **(A**) EGFR TKI sensitive cells are EGFR signal dependent in survival and proliferation. **(B)** EGFR and AXL collectively regulate survival and proliferation in acquired EGFR TKI resistant cells. **(C)** A switch from an EGFR pathway dependent signal transduction pattern to an AXL/MET complex dependent pattern plays critical roles in acquired EGFR TKI resistance correlated EMT. AXL/MET over-expression, GAS6/HGF up-regulation, MET amplification, and AXL/MET might mediate uncoupling of EGFR activity from its kinase function and are reported causes of this switch to EGFR signaling independence.

In addition to AXL overexpression, MET overexpression and the expression of its ligand, HGF have both been described as in vitro and in vivo mechanisms of resistance to EGFR TKI therapy in EGFR-mutated lung cancers[113]. The AXL and MET kinases share a number of structural features and similarly play important roles in cell migration, invasion and metastasis. Recent elegant studies shed some light on the biochemical interplay of these pathways [41, 114, 115]. In a study set out to define the spectrum and mechanism of HGF/MET-mediated resistance, it was noted that HGF-induced EGFR TK inhibition is a very common mechanism in human cancers and in such cases the kinase-inactive EGFR directly interacts with and stabilizes several cancer-relevant proteins, including AXL, EphA2 and the Cub domain containing protein 1. Overall, the model shows that HGF/MET activation can uncouple the oncogenic activity of EGFR from its tyrosine kinase activity, effectively allowing downstream signaling activation via aggregation and transmitting of EGFR activation to AXL and other receptor kinases (Figure 2). In fact, HGF activity could block phosphorylation of EGFR by ligand stimulation mediated via HGF/MET activation of MEK: furthermore MEK inhibitors can reverse this phenomenon. This observation of an alternative mode of EGFR action may explain why despite the continued inhibition of EGFR TK activity by EGFR TKIs, downstream activation of PI3K and other pathways can persist in the presence of MET/AXL co-expression. The binding capacity of the interacting partners, AXL, CDCP1 and EphA2 were unaltered by gefitinib treatment but binding was effectively abrogated by MET TK inhibition and MET TK therapy led to restoration of the anti-proliferative effects of gefitinib. It remains to be defined whether AXL overexpression alone in the above listed resistant models can “make up” to some extent for the lack of HGF/MET activity and provide a weaker alternative uncoupling of EGFR activity [116]. However, these studies clearly define MET and AXL as key targets in EGFR/ErbB2-resistance and also highlight the potential for novel therapies interfering with the complex interactions at the cell membrane interface leading to aggregation of these disparate receptors (Figure 2).

## AXL/MER as immunosuppressive RTKs: a cautionary note for cancer therapy

Deficiencies in TAM signaling have been shown to play key roles in sustained immune activation and chronic inflammation raising some concerns about the use of AXL inhibitors in cancer therapy. For example, in many cells, some cytokine receptor signaling systems, such as signaling via the type 1 interferon receptor, are co-dependent on TAM family receptors [117]. AXL and MER have indeed been demonstrated to be expressed on multiple immune cells, such as dendritic cells and macrophages and have an essential immune-suppressive role via the abrogation of Toll-like receptor and cytokine receptor signaling. In the inflammation-associated colorectal cancer model by azoxymethane and dextran sulfate sodium-induced inflammation in mice, lack of AXL and MER led to increased production of inflammatory cytokines favoring a tumor-promoting environment leading to the enhanced formation of colonic adenomatous polyps [118]. Therefore, the potential for adverse effects for tumor promotion through the use of systemic anticancer therapies targeting AXL might be a concern and this highlights the importance of more thorough understanding of the tissue and cell-type specific functions of AXL. The development of targeting strategies devoid of pro-inflammatory adverse effects might be optimal for future development. Therefore, treatment strategies that spare the RTKs in the macrophage compartment will be relevant – e.g. therapeutics targeting the unique interface between AXL and EGFR/MET could provide such selective advantages.

On the other hand, another recent study suggests a reverse role for TAM receptors in the regulation of cancer metastases by the modulation of NK cell activity. This study found that mice with targeted inactivation of the E3 ubiquitin ligase, Cbl-B demonstrate enhanced NK cell activity against metastatic tumor models and this unique activity was found to be related to the regulation of TAM family receptors that were found to be ubiquitylation substrates for Cbl-b. Indeed, further studies showed that a small molecule TAM kinase inhibitor, LDC1267 confers treatment potential through enhancing in vivo NK cell activity and very interestingly, this activity is mimicked by the anticoagulant warfarin which through its gamma-carboxylase inhibitor activity leads to the generation of deficient Gas6 unable to activate TAM receptors. These key observations suggest that inhibition of TAM/AXL activity could in fact have additional anti-cancer activity through NK cell potentiation against metastatic disease [119].

## Inhibition of AXL as a cancer treatment strategy

As highlighted before, an overexpressed/overactivated Gas6-AXL axis in a wide variety of cancers can contribute to their progression, including invasion, metastasis, and resistance both to chemo as well as targeted anti-cancer therapies, indicating that the Gas6/AXL pathway is a very promising target for cancer therapy. Inhibition of this pathway can practically be achieved through multiple means e.g. by using AXL TKIs, drugs targeting the AXL/MET/EGFR interface, and their downstream signaling effectors or by using blocking antibodies against Gas6 and AXL. As shown in Table 2 multiple drug companies are pursuing the clinical development of a variety of inhibitors either specifically against AXL or multi-targeted kinase inhibitors with good potency against AXL as well.

**Table 2:** AXL inhibitors under development

Compound	Known Targets	Clinical Trials.gov identifier	Phase of Development	Sponsor	Functions in a disease indication and other preclinical research details	References
**Small Molecule Inhibitors**						
LY2801653	AXL, MET, MST1R	NCT01285037	Phase 1	Eli Lilly and Co.	An orally bioavailable multi-kinase inhibitor against MET, AXL, MST1R.	[120, 121]
MP-470 (Amuvatinib)	AXL, c-KIT, PDGFR, FLT3, RET, RAD51	NCT00894894 NCT00881166 NCT01357395	Phase 1 Phase 1 Phase 2	Astex Pharma.	c-Kit/AXL tyrosine kinase inhibitor investigated in stromal tumors and in breast cancer.	[102]
SKI-606 (Bosutinib)	AXL, Src Kinase, Abl, TGFB, BMP	NCT00195260 NCT00319254	Phase 1 Phase 2	Pfizer	Treating HCC cells with Bosutinib decreased the AXL specific invasiveness of HCC cell lines.	[122]
MGCD 265	AXL, MET, VEGFR	NCT00697632 NCT00975767	Phase 1 and 2 Phase 1	Mirati Inc.	Phase 2 NSCLC	[123–126]
MGCD516	MET, AXL, RET, TRK, DDR2, KDR, PDGFRA, or KIT		Preclinical	Mirati Inc.	Phase 1 planned	[139]
ASP2215	AXL, Flt3	NCT02014558	Phase 1 and 2	Astellas Pharma.	A novel FLT3/AXL inhibitor: Preclinical evaluation in acute myeloid leukemia	[110]
XL184 (Cabozantinib)	AXL, c-MET, VEGFR-2, c-KIT, Flt 1/3/4, Tie2 and RET	NCT01639508	Phase 2 and 3	Exelixis	Medullary Thyroid cancer, Brain cancer, NSCLC and randomization discontinuation trial in various solid tumors	[127–130]
BMS-777607 (ASLAN 002)	AXL, Mer and MET	NCT01721148	Phase 1	Aslan Pharma. and Inventive Health Clinical	Selective small molecule kinase inhibitor against AXL, Mer, and Met.	[131]
GSK1363089/XL880 (Foretinib)	AXL, cMET, VEGFR2	NCT02034097	Phase 2	GlaxoSmithKline	Restores lapatinib sensitivity in lapatinib-resistant breast cancer cells with AXL over expression.	[101]
SGI-7079	AXL	NCT00409968	Phase 2	Astex Pharma	Decreased malignant properties in inflammatory breast cancer. Combination of SGI-7079 with erlotinib reversed erlotinib resistance in mesenchymal cell lines, xenograft model of mesenchymal NSCLC and patients.	[43, 93]
S49076	AXL, MET, EGFR	ISRCTN00759419	Phase 1	Servier	Preclinical activity in colon carcinoma.	[132]
R428 (BGB324)	AXL	European Clinical trial	Phase Ia	BerGen BIO	Resensitized HN5-ER cells to erlotinib in head and neck cancer, reduced migration and invasion in melanomas, induced CLL B-cell apoptosis, reduced invasion and migration in EAC cell lines, reduced metastatic burden and extended survival in metastatic breast cancer.	[21, 133]
DP3975	AXL		Preclinical	Deciphera Biotech	Inhibited cell migration and proliferation in mesotheliomas.	[140]
NPS-1034	AXL, MET		Preclinical	NeoPharma	Newly developed drug that targets both MET and AXL in NSCLC cells with acquired resistance to gefitinib or erlotinib.	[106]
LDC1267	AXL, Tyro, Mer		Preclinical		Induces NK cells to kill tumor cells in mouse metastatic breast cancer and melanoma model	[119]
NA80x1	AXL		Preclinical		Inhibits AXL phosphorylation, cell motility, and invasion in MDA-MB-435 cells.	[141]
**Receptor Monoclonal Antibody**						
YW327.6S2	AXL		Preclinical		Anti-AXL monoclonal antibody.	[134]
**Nucleotide Aptamer**						
GL21.T	AXL		Preclinical		Binds to the extracellular domain of AXL to inhibit its catalytic activity in lung cancer.	[135, 136]

Many of the ongoing clinical trials of such multikinase drugs have been initiated without a current focus on AXL as a main target. As early phase studies of such compounds are completed and AXL continues to emerge as a promising target, studies are starting to emerge with a specific focus on AXL. Some more relevant studies are listed below. For example, LY2801653 is a type-II ATP competitive multi kinase inhibitor of MET and AXL and MST1R that has been shown to inhibit cellular functions of MET, including cell migration and proliferation and anti-tumor activities in multiple mouse xenograft models [120] [121]. Phase I studies of LY2801653 are being pursued in patients with advanced cancer. MP470 (Amuvatinib), another multi kinase inhibitor targeting AXL is being developed actively and is being studied specifically in gastrointestinal stromal tumors and breast cancer [102]. Reduction in AXL specific invasiveness in Hepatocellular carcinoma cells has been observed with another multikinase inhibitor SKI606 (Bosutinib) [122]. SKI606 (Bosutinib) is now in Phase 1 and 2 clinical trials for patients with advanced solid tumors including metastatic breast cancer. Two other exciting compounds with excellent potency against AXL, MGCD265 and MGCD516 are in the initial phases of clinical development, including in Non small cell lung cancer (NSCLC) [123–126]. ASP2215, which besides showing excellent activity against FLT3, also is potent against AXL is in phase 1/2 stages of clinical development in relapsed and refractory AML patients[110]. XL184 (Cabozantinib), another multi kinase inhibitor including AXL is in phase 2 and 3 of clinical trials against many different types of cancers, such as medullary thyroid, brain and NSCLC cancers [127–130]. BMS-777607, another selective small molecule kinase inhibitor against AXL, Mer, Tyro3 and Met is in Phase 1 studies in subjects with advanced or metastatic solid tumors [131]. GSK1363089, also known as XL880 (Foretinib), an oral multi-kinase inhibitor targeting MET, RON, AXL has been shown to restore sensitivity in lapatinib-resistant HER2 positive breast cancer cells with AXL overexpression [101]. XL880 is currently in several clinical studies, including a phase II, open label, uncontrolled, parallel, multi-cohort, multicenter 2-stage study to assess the safety and efficacy in NSCLC patients. Another highly specific AXL inhibitor, SGI-7079 has led to decreased malignant properties in inflammatory breast cancer in preclinical studies. Combining SGI7079 with erlotinib reversed the resistance in different model systems, such as in mesenchymal cell lines and xenograft models of mesenchymal NSCLC [43, 93]. Based on these studies SGI7079 is now in phase 2 studies. S49076 is a novel ATP-competitive tyrosine kinase inhibitor of MET, AXL/MER, FGFR1/2/3 that can inhibit autophosphorylation of these RTKs and their downstream signaling in vitro/vivo. On the basis of preclinical activity in colon carcinoma, a phase I trial of S49076 has been initiated in Europe [132]. R428 (BGB324) is another novel small-molecule inhibitor that can potently block autophosphorylation of AXL on the COOH-terminal multiple docking site Tyr821 and consequent activation of AKT, SFK phosphorylation with low nanomolar affinity [21]. R428 has entered phase I clinical trials in 2013 [133]. In addition to the above experimental drugs there are some more mentioned in the table which on the basis of lab findings seem to be very potent to enter into clinical trials sometime in future.

Other novel methods of inhibiting AXL receptor specifically include an anti AXL monoclonal antibody, YW327.6S2 which has shown highly specific activity in preclinical models[134]. Another quite novel approach is the development of the Nucleotide aptamer, GLT21.T which binds to the extracellular domain of AXL and inhibits its activity in cellular models of lung cancer [135, 136].

Another somewhat different route of targeting AXL overexpression/activity might be through the inhibition of key proteins involved in the regulation of protein trafficking called chaperones, such as heat shock proteins (HSP’s) involved in protein processing. Recently AXL in fact was shown to be a client protein of the hsp90 chaperone. In an AXL-dependent thyroid cancer model system, treatment with the hsp90 inhibitor, 17-AAG led to a substantial reduction in AXL expression accompanied by downregulation of AXL activation and downstream signaling activity. Elegant studies demonstrated that this activity is dependent on modulation of the trafficking of the fully glycosylated, mature 140 kDA form of AXL and this phenomenon appears independent of AXL phosphorylation. Downregulation in the presence of hsp90 inhibition is mediated by proteasomal degradation as a result of polyubiquitination through binding of the hsp70/CHIP E3 ligase. These data strongly suggest that AXL overexpression and activity could be successfully targeted in AXL-addicted tumors or in AXL-mediated drug –resistant cancers via hsp90 inhibition [137].

## Biomarker development for AXL-targeting agents

One key issue for the development of AXL-targeted therapies for human use is the selection of appropriate biomarkers for patient enrichment. As no genetic changes have been reported so far correlating with AXL overexpression, IHC appears to be the method of choice and has been utilized in a number of in vitro and animal studies successfully. Another potentially appealing biomarker for clinical studies might be imaging AXL expression in vivo using labeled probes. One study showed successful labeling with the use of an anti-human monoclonal AXL antibody radiolabelled with I^125^ via SPECT/CT imaging in mice xenografted with AXL-high and AXL-low pancreatic and prostate cancer cell lines and uptake appeared to correlate closely with IHC findings. As AXL expression can be variable within tumors and as acquired drug resistance studies suggest that it can change temporally, a biomarker that can image AXL expression spatially as well as over time would be highly desirable and with positron-emitting isotopes, such as ^89^ZR might be amenable to immuno-PET imaging which could be readily incorporated into AXL-targeting clinical studies [138].

## Future perspectives

The last few years have seen tremendous advances in our understanding of the wide relevance of AXL activation in multiple aspects of oncogenesis, invasion, metastasis and drug resistance. The field appears poised to take on the next and most important task and define the true translational potential of AXL inhibition in the clinic. Key issues to address in the coming years include the following relevant questions:

1. What is the mechanism of AXL overexpression/activation in human cancers? Is AXL overexpression mediated through genetic, epigenetic or other mechanisms in different cancer subsets and could better understanding of AXL regulation yield clinically useful biomarkers?
2. What is the correlation between AXL expression and the elusive EMT phenomenon? Is AXL a driver or an effector of EMT or is it just an epiphenomenon of the EMT process?
3. How do the AXL/MET cell surface complexes form in drug resistant tumors and could better understanding of this key bypass pathway yield novel therapeutics to affect drug resistance?
4. How does AXL inhibition impact cancer immunity outside of its utility to block tumor metastasis, invasion and reverse drug resistance? Can immune deficiency be a major roadblock in the development of AXL inhibitors or can it be bypassed through some means? Alternatively, could inhibitors of the AXL/GAS6 axis have beneficial anti-cancer effects through NK cell activation?
5. Clinical studies of AXL inhibitors will need to incorporate multiple potentially relevant biomarkers of AXL activity/dependency as well as biomarkers of anti-tumor immunity to ensure that key signals from ongoing studies are not missed in this area which currently lacks a validated biomarker for appropriate patient selection. In addition, given the very different contextual aspects of AXLs role in carcinogenesis, invasion/metastasis/EMT and drug resistance, clinical studies will need to assess these carefully in each particular scenario.

## ABBREVIATIONS

AML: Acute myeloid leukemia
RTK: Receptor tyrosine kinase
TAM: RTK subfamily that includes TYRO-3, AXL, MER
EMT: Epithelial–mesenchymal transition
Gas 6: Growth arrest specific gene 6
MZF1: Myeloid zinc finger 1
NRTK: Non-receptor tyrosine kinase
NSCLC: Non-small cell lung cancer
ROS: Reactive oxygen species
TKI: Tyrosine kinase inhibitor
TNBC: Triple negative breast cancer
Tulp-1: Tubby-like protein 1
YAP1: Yes-associated protein 1
